# Additive Manufacturing of Lithium Disilicate with the LCM Process for Classic and Non-Prep Veneers: Preliminary Technical and Clinical Case Experience

**DOI:** 10.3390/ma15176034

**Published:** 2022-09-01

**Authors:** Alexey Unkovskiy, Florian Beuer, Dilan Seda Metin, Daniel Bomze, Jeremias Hey, Franziska Schmidt

**Affiliations:** 1 Department of Prosthodontics, Geriatric Dentistry and Craniomandibular Disorders, Charité-Universitätsmedizin Berlin, Corporate Member of Freie Universität Berlin, Humboldt-Universität zu Berlin, Aßmannshauser Str. 4–6, 14197 Berlin, Germany; 2 Department of Prosthodontics, Peoples’ Friendship University of Russia (RUDN University), 117198 Moscow, Russia; 3 Lithoz GmbH, Mollardgasse 85a/2/64-69, 1060 Vienna, Austria

**Keywords:** vat polymerization, 3D printing, digital workflow, laminate veneers, non-prep veneers, dental ceramics

## Abstract

Background: ceramic veneers, crowns, and other types of restorations are often made using either the press heating technique or the subtractive method. The advent of lithography-based ceramic manufacturing (LCM) allows for the manufacturing of such restorations in an additive way. Methods: this concept paper describes the first clinical experience in the application of LCM lithium disilicate restorations in vivo for the manufacturing of classic veneers for a patient with severe tooth wear. The applied restorations were analyzed in terms of their marginal fit in metrology software (Geomagic Control X, 3D Systems). Furthermore, the feasibility of 3D printing of non-prep veneers with a 0.1 mm thickness was tested. Results: the classic LCM lithium disilicate veneers were tried in the mouth cavity and demonstrated adequate esthetics and a sufficient marginal fit of 100 µm. Furthermore, the non-prep veneers with a 0.1 mm thickness could be successfully printed using LCM technology and also demonstrated an adequate fit on the model in vitro. Conclusions: the described technical approach of lithium disilicate 3D printing with LCM technology may pose a valid alternative to subtractive and analog manufacturing and be a game-changing option with the use of additive chairside ceramic fabrication.

## 1. Introduction

Dental glass-ceramic, especially lithium disilicate, is widely used for the manufacturing of crowns, overlays, and veneers in the esthetically relevant region as it overcomes the limitations of metal-ceramic restorations [1]. The advent of digitization and computer-aided design and manufacturing (CAD/CAM) in the dental field provided new technical and clinical protocols for the design and manufacturing of ceramic restorations [2]. Even though there is not sufficient evidence on the superior performance of the digital workflow in comparison to conventional approaches of dental ceramics fabrication [3], it has been widely applied in the clinical practice in recent years [4,5]. Whereas the semi-digital workflow allows for the additive manufacturing of wax restoration prototypes, the fully digital workflow allows for the computer-aided manufacturing of the final ceramic restoration using milling technologies [6]. However, subtractive manufacturing does not provide the absolute freedom in its design as there may be some applications that cannot be achieved by milling, due to being unattainable for a milling instrument [7]. In such cases, additive manufacturing, with its layer-by-layer working principle, surpasses the subtractive one. Some pilot studies proved the feasibility of the fabrication of dental ceramics in an additive approach, such as yttria-stabilized zirconia (ZrO_2_), alumina (Al_2_O_3_), and tricalcium phosphate (TCP) [8,9]. The limitations of such manufacturing processes relate mostly to the ceramics’ post-processing, which significantly extends the average manufacturing time, making it less attractive for the clinical practice. In contrast, as demonstrated at the Technische Universität Wien (TU Wien), lithium disilicate may qualify for the additive chairside clinical application as it utilizes a more rapid stereolithographic ceramic manufacturing (LCM) process [10]. Baumgartner et al. reported that lithium disilicate restorations manufactured with LCM showed adequate printing accuracy and mechanical properties and can be manufactured within one day [11]. However, the literature lacks any clinical reports on the application of additively manufactured ceramic restauration in the in vivo environment.

The clinical application of ultra-thin laminate veneers and occlusal veneers may pose a valid alternative to conventional crowns as they can also provide a sufficient bearing capacity [12,13]. This can be attributed to the fact that no preparation or minimal preparation allows for direct bonding to the enamel, providing a greater bonding strength [14]. As subtractive manufacturing of thin ceramic parts may be prone to fracture, it may not be the best option for the manufacturing of laminate veneers and non-prep veneers, which often do not exceed a thickness of 0.3 mm [15,16,17]. Therefore, such restorations are still most commonly produced in an analogue workflow [18]. The LCM technology allows the manufacturing of thin restorations from lithium disilicate in the span of 0.1–0.2 mm and can, therefore, substitute the heat pressing technique.

The present clinical case demonstrates the preliminary experience with the clinical application of lithium disilicate restorations in the anterior region produced by LCM. Furthermore, a pilot trial was performed with the additive manufacturing of thin non-prep veneers on a phantom model.

## 2. Materials and Methods

### 2.1. Clinical Presentation of Classic Venners

**Figure 1:**
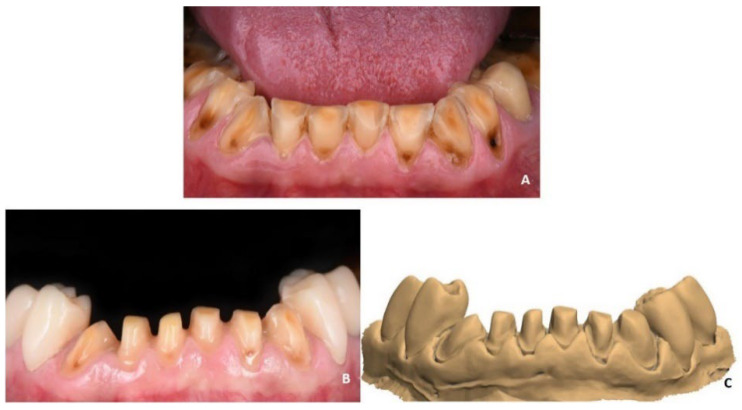
(**A**) initial situation with severe wear; (**B**) preparation of six frontal teeth with chamfer in the vestibular and proximal areas; (**C**) digital impression obtained with intraoral scanner (IOS).

A 60-year-old female patient with severe wear in the whole dentition (Figure 1A) was referred to the Department of Prosthodontics at the Charité University Hospital and received long-term temporary composite restorations in the lower jaw for the reestablishment of the vertical dimension of occlusion (VDO). She also gave her informed written consent for the manufacturing of six lithium disilicate veneers in the anterior area using the LCM technology. The six anterior teeth in the lower jaw were prepared with a 0.5 mm chamfer in the vestibular and proximal areas (Figure 1B). The gingival retraction was performed using the two-cord technique. An intraoral scanner (IOS), Trios 4 (3shape, Copenhagen, Denmark), was used to capture the upper and lower jaws with the final VDO in the standard tessellation language (STL) format (Figure 1C). Two buccal scans were performed for the bite registration.

**Figure 2:**
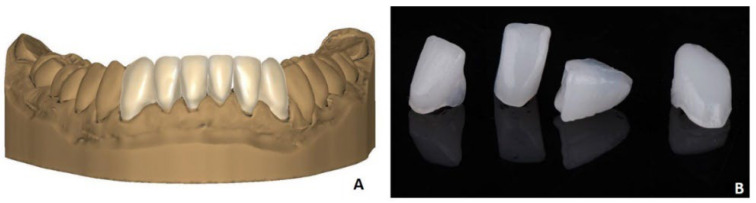
(**A**) Six frontal restorations designed in DentalCAD software; (**B**) additively manufactured veneers with LCM technology directly after printing.

Six veneers were designed in the CAD software (DentalCAD, version Galway, Exocad, Darmstadt, Germany) and exported as STL data (Figure 2A). These were uploaded in the data preparation software CeraFab Control (version 8, Lithoz, Vienna, Austria) and printed with a CeraFab System S65 Medical (Lithoz, Vienna, Austria). The printer uses the principle of vat photopolymerization with mask exposure, which is also known as DLP (digital light processing). In conjunction with highly-filled ceramic suspensions of photocurable resins, this process is called lithography-based ceramic manufacturing (LCM) and allows the production of dense and precise ceramic products. Labial-placed support structures were generated with the Software Deskartes 3D Data Expert (Deskartes Oy, Helsinki, Finland) and loaded in the data preparation software of the printers alongside the STL of the restorations (Table 1). After printing the LCM-printed lithium disilicate, restorations were post-processed as follows. The green parts (still containing organic binder) were cleaned from excess slurry with the CeraCleaning Station Ultra (Lithoz, Vienna, Austria) with the proprietary cleaning fluid LithaSol 30 (Lithoz, Vienna, Austria). Subsequently, the restorations were placed in a furnace (L40, Nabertherm, Lilienthal, Germany) and heated up to 430 °C, followed by a dwell time of 6.5 h. The final sintering was conducted within a Programat CS3 (Ivoclar Vivadent GmbH, Ellwangen, Germany) at a temperature of 900 °C with a dwell time of 1 sec. Finally, support structures were removed with a laboratory handpiece (Perfecta 900, W & H, Bürmoos, Austria) (Figure 2B).

**Figure 3:**
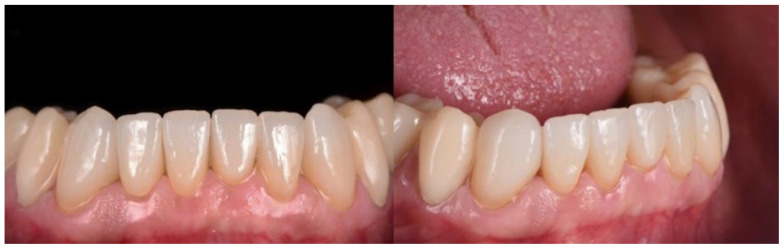
The LCM-manufactured lithium disilicate restorations in situ.

The post-processed restorations in a generic color were customized using an extrinsic coloring set (Ivocolor, Ivoclar Vivadent, Ellwangen, Germany). The final restorations were tested on the patient using try-in gel (Variolink Esthetic try-in-paste, Ivoclar Vivadent, Ellwangen, Germany) (Figure 3).

**Figure 4:**
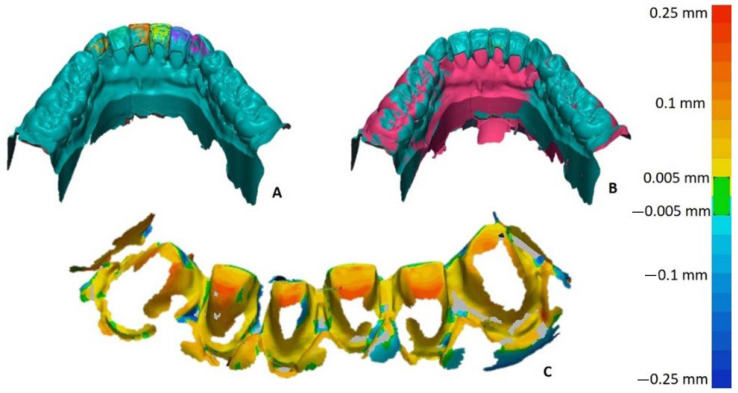
(**A**) A scan of each single restoration (multicolor) with the interior surface matched with the scan of restorations in situ (blue); (**B**) scan of the restorations in situ (blue) matched with the scan of the preparation (red); (**C**) heat map of the distances between the interior surface of each LCM restoration and corresponding preparation surface, representing the bottom adaptation.

In order to assess the marginal adaptation on the dyes, all six restorations in situ were scanned again with an IOS. Furthermore, the interior surface of each single restoration was scanned with a laboratory scanner (D2000, 3 Shape, Copenhagen, Denmark). The single scans of each restoration were matched with the scan of the same restorations in situ in the metrical software (Geomagic Control X, 3D Systems, Morrisville, NC, USA) using the best fit function (Figure 4A). The teeth preparation scan was also aligned with the scan of restorations in situ (Figure 4B). The marginal and bottom adaptation of the LCM-printed restorations were assessed according to the distance between the interior surface of each restoration and preparation surface of the corresponding dye (Figure 4C).

The LCM-printed restoration was not cemented as the printed lithium disilicate had not yet received approval for definitive restorations, which is planned in the near future.

### 2.2. Non-Prep Veneers

**Figure 5:**
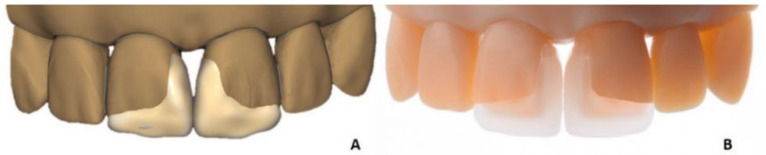
(**A**) virtual design of non-prep veneers for diastema closure; (**B**) LCM-manufactured non-prep veneers with the minimal thickness of 0.1 mm.

A typodont model of simulated diastema in the upper jaw was scanned with an IOS. Two restorations were designed in DentalCAD software with a minimal thickness of 0.1 mm (Figure 5A). The STL file of each restoration was sent to the CeraFab System S65 Medical 3D printer and printed with lithium disilicate in the generic color analog to the classical prep veneers as described previously. The supporting structures were placed on the proximal surfaces. The printing time depended on the height of the restoration and the chosen layer time—in this case, the total printing time was around 5 h. After cleaning, debinding, and sintering, the supports were removed manually, as described above. The restorations were tried in the model (Figure 5B).

## 3. Results

The performed bottom adaptation analysis revealed distances of 100 µm on most of the preparation surfaces with areas of 150 µm on the peak of each dye. The adaptation distance showed a decreasing tendency towards the marginal area. Clinically, the LCM classic veneers demonstrated an adequate marginal seal, which was discovered optically and by probing. The LCM non-prep veneers were tried in the model and also demonstrated an adequate marginal seal, which could be seen only optically.

## 4. Discussion

The presented proof-of-concept case demonstrates 3D printing of classic and non-prep veneers using LCM technology. This technology has previously been shown to produce accurate parts with the maximum deviation of 50 µm to the STL-file [11]. The performed bottom adaptation analysis revealed distances of 100 µm on most of the preparation surfaces with areas of 150 µm on the peak of each dye. The adaptation distance showed a decreasing tendency towards the marginal area. These parameters correlate with the accuracy of heat-pressed and milled restorations [19]. The demonstrated marginal adaptation of LCM veneers may be regarded as a clinical success [20]. The fact that examined restorations were not cemented might not have negatively influenced their marginal fit [21]. The applied method to evaluate the bottom adaptation may include the matching bias as the scanning of each single restoration as well as the fitted restorations within the mouth cavity may be prone to inaccuracies. For this reason, systematic research is needed to provide evidence on the fit of LCM restorations.

The finding of this proof-of-concept clinical case coincides with the data obtained by Baumgartner et al., showing the most dimensional changes in the middle of the LCM restoration [11]. Schönherr et al. described the frame-like supporting structure to be optimal for LCM and highlighted which areas of the restoration are most suitable for placement of supporting structures [22]. However, these recommendations are sometimes hard to follow in the case of minimally invasive restorations as the complex geometry of interior and proximal surfaces may influence the placement of supporting structures. Further optimization of the build angle for such fragile ceramic restoration may be investigated in future research.

**Figure 6:**
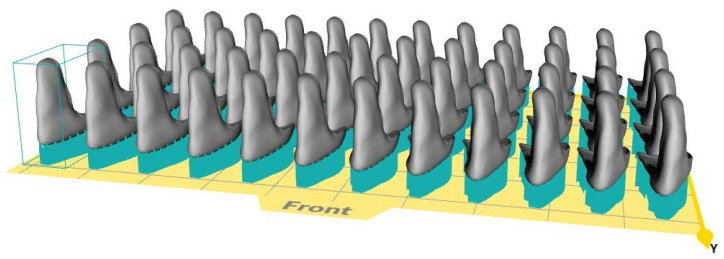
Diastema non-prep veneers on a support structure placed on the virtual build platform of a CeraFab System S65 Medical 3D printer.

With regards to production time, up to 48 pieces of the displayed diastema veneers can be placed at once on a build platform of a CeraFab System S65 Medical (Figure 6). The print run of a full platform takes a little less than 5 h, resulting in a printing time per part of around 6 min. The post-processing for each part can be conducted in around 2 min, including the placement in the furnaces. The time needed for individualization and coloring of the post-processed parts is not included as it highly depends on the skills of the dental technician and the demands of the patient.

The future perspective of additive manufacturing of lithium disilicate may also evolve to multicolor 3D printing. This would be a game-changing feature as it would allow omitting the individualization by the dental technician and would bring us one step closer to full chairside lithium disilicate manufacturing.

With regards to the price of the final 3D printed lithium disilicate restoration, the cost estimation is mostly based on the material price, the machine depreciation, and the costs for post-processing manual labor.

The current proof-of-concept clinical case does not consider the mechanical and adhesive properties of printed restorations. Thus, flexural and bonding strength should be assessed in future research alongside the surface characterization of the LCM-manufactured lithium disilicate. The demonstrated clinical and technical approaches also open new horizons for in vivo testing of such additively manufactured ceramic restorations as soon as this material, in combination with the printing technology, receives the approval for its clinical application.

## 5. Conclusions

The current concept paper presents the first clinical experience with LCM-manufactured classic veneers. Furthermore, it demonstrates the technical feasibility of additive manufacturing of thin ceramic non-prep veneers. The described technology seems to be a viable option for the chairside manufacturing of lithium disilicate restorations and may pose a game-changing alternative to the subtractive and heat-pressing technologies in the future. Aystematic research is needed in the future to provided evidence on the clinical performance of LCM restorations.

## Figures and Tables

**Table 1:** Printing parameters for lithography-based ceramic manufacturing on a CeraFab System S65 Medical for lithium disilicate anterior teeth veneers.

Ceramic material	Lithium disilicate generic color with 45 Vol% solid loading and chemical composition based on IPS e.max^®^ Press LT (Ivoclar Vivadent AG, Liechtenstein)
Layer height	25 µm
Number of layers	606
Layer time	36 s
Runtime for whole print run	6 h
Exposure intensity starting layers	200 mJ/cm^2^
Exposure intensity general layers	175 mJ/cm^2^
Lateral (XY) shrinking compensation	1.31
Build direction (Z) shrinking compensation	1.35
Z curing depth compensation	Off
Z curing depth compensation layers	0
Contour offset	0 µm
Support structure thickness	380 µm
Vat type	Ultra-High-Contrast (UHC) with CeraVat F
Cleaning fluid	LithaSol 30

## Data Availability

Not applicable.
